# Case Report: Dubin-Johnson Syndrome Presenting With Infantile Cholestasis: An Overlooked Diagnosis in an Extended Family

**DOI:** 10.3389/fped.2022.855210

**Published:** 2022-05-25

**Authors:** Naglaa M. Kamal, Omar Saadah, Hamdan Alghamdi, Ali Algarni, Mortada H. F. El-Shabrawi, Laila M. Sherief, Salma A. S. Abosabie

**Affiliations:** ^1^Department of Pediatrics and Pediatric Hepatology, Faculty of Medicine, Cairo University, Cairo, Egypt; ^2^Department of Pediatrics, Faculty of Medicine, King Abdulaziz University, Jeddah, Saudi Arabia; ^3^Department of Pediatrics, Alhada Armed Forces Hospital, Taif, Saudi Arabia; ^4^Department of Pediatrics, Taif Children Hospital, Taif, Saudi Arabia; ^5^Faculty of Medicine, Zagazig University, Zagazig, Egypt; ^6^Faculty of Medicine, Julius-Maximilians-Universität Würzburg, Würzburg, Germany

**Keywords:** Dubin-Johnson syndrome, infant, cholestasis, ABCC2 gene, mutation

## Abstract

Dubin-Johnson syndrome (DJS) is an often-missed diagnosis of neonatal cholestasis. We report two patients with DJS, who presented with neonatal cholestasis. The first patient underwent extensive investigations for infantile cholestasis with no definitive etiology reached; the diagnosis of DJS was missed until the age of 14 years old. The diagnosis was confirmed genetically with c.2273G > T, p.G758V mutation in exon 18 of the ABCC2 gene. The 2nd patient is a 7-day-old baby, the son of the 1st patient who gave birth to him at the age of 21 years old. He was diagnosed with DJS at the age of 2 weeks based on normal clinical and laboratory workup apart from direct hyperbilirubinemia. He had the same mutation as his mother in homozygous status. The husband was heterozygous for the same mutation. DJS is one of the often-missed differential diagnoses of neonatal cholestasis. It should be suspected in patients of infantile cholestasis, who have an, otherwise, normal physical examination, and laboratory investigations to avoid unnecessary lengthy, invasive, and expensive workups.

## Introduction

Dubin-Johnson syndrome (DJS) was first reported in 1954 by Dubin and Johnson (1) as a rare autosomal recessive disease with clinical features of chronic-conjugated hyperbilirubinemia due to a defect in the excretion of the anionic conjugate from the hepatocytes into the bile (2). Most patients manifest as intermittent or chronic jaundice aggravated by intercurrent illness (1). Physical examination is frequently unremarkable (1). Liver enzymes are usually within normal limits, while bilirubin levels fluctuate (1).

The syndrome occurs due to expression defects of the MRP2 gene, an ATP-dependent canalicular membrane transporter (3–5). The diagnosis is established by performing the bromsulphalein test, oral cholecystography, HIDA scan, and liver biopsy (6–8). Liver biopsy is the gold standard diagnostic test for this syndrome. It shows the presence of brown pigment granules in the centrilobular hepatocytes (9–11). Molecular genetic testing of the ABCC2 gene is the definitive diagnosis (12). We, herein, report a Saudi female child who presented as having cholestasis at the age of 1 month with a missed diagnosis of DJS until the age of 14 years. Her molecular genetic testing revealed the c.2273G > T, p.G758V mutation in Exon 18 of the ABCC2 gene.

## Case Report

A 14-year-old female child was born to consanguineous Saudi first-degree cousins who are descents from a tribe with highly consanguineous marriage. She was referred to a pediatric gastroenterology clinic as a case of persistent conjugated hyperbilirubinemia for investigations.

Tracing her history revealed that jaundice started at the age of 4 days with elevated total bilirubin (350 μmol/L), mainly indirect. Her direct bilirubin was 30 μmol/L with normal alanine and aspartate transaminases (ALT and AST). Phototherapy was started, and the patient was discharged in good condition after 3 days.

She returned to her primary physician at the age of 40 days with unresolved jaundice and mild abdominal distension with no organ enlargement. Her investigations revealed mild direct hyperbilirubinemia. Her total and direct bilirubins were 50 and 35 μmol/L, respectively. Her ALT, AST, gamma-glutamyl transpeptidase (GGT), prothrombin time/concentration, and abdominal ultrasonography were all normal. Extensive workups of cholestasis, including complete blood count, retics, coombs, hemoglobin electrophoresis, urine and blood cultures, TORCH screening, serum bile acids, thyroid profile, tandem metabolic screening, and non-glucose-reducing substances in the urine, were all normal. HIDA scan and MRCP were not available in that hospital and were not done. Liver biopsy was refused by the parents, and she was discharged against medical advice in good general condition without a definitive diagnosis. Her total and direct bilirubin at time of discharge was 48 and 40 μmol/L, respectively.

Since that time and until her presentation to our care at the age of 14 years, the parents used to visit different health care facilities when their child’s jaundice deepened with different intercurrent illnesses. Laboratory workups, including liver function tests and hepatitis markers, were done many times with normal results apart from direct hyperbilirubinemia.

On presentation to our hospital, she had tinge jaundice with stable vital signs, normal abdominal examination with no organomegaly, and normal assessment of different body systems. Her laboratory investigation showed high total bilirubin of 32 μmol/L, mainly in the form of direct bilirubin (31 μmol/L), with normal ALT, AST, GGT, complete blood picture, and a renal profile with normal abdominal US. The diagnosis of DJS was suspected, and a 99*^m^* Tc-HIDA scan was requested. The HIDA scan serial images revealed rapid clearance of blood pool activity with a good hepatocyte function as evidenced by the adequate ascending limb of the dynamic curve. However, there was a slow excretion of radioactivity into the biliary radicles with retained activity in the liver up to 6 h. The gall bladder was seen at 1 h and the small intestine at 2 h. This good hepatocyte uptake function with impairment of excretory function in absence of obstruction was highly suggesting DJS. Urinary coproporphyrins were not done (the test was not available in our hospital).

Molecular genetic testing for DJS, the ABCC2 gene, was requested to confirm the diagnosis.

## Molecular Genetic Analysis of the ABCC2 Gene

PCR amplification and direct sequencing of all coding exons and flanking intronic sequence (ABCC2 gene, GenBank NM_000392.3, NC_000010.10) gene dosage analysis by quantitative real-time PCR (qPCR) with 5 amplifications (in exons 1, 7, 15, 24, and 32) (13).

## Results

**FIGURE 1:**
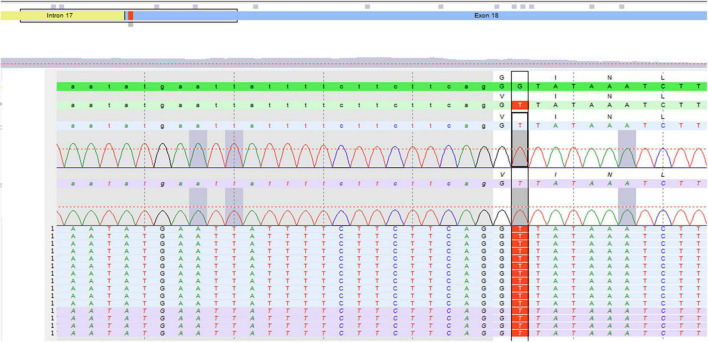
Molecular genetic testing of the patient.

Unclassified variant c.2273G⟩T, pG785V in Exon 18 of the gene ABCC2 gene in the homozygous state. By qPCR, no deletion or duplication was detected, Figure 1.

## Interpretation

Molecular analysis confirmed the clinical suspicion of DJS syndrome. The variant c.2273G⟩T, pG785V in Exon 18 of the gene ABCC2 gene was detected in homozygous state.

The patient was diagnosed in 2014, and, at that time, this mutation was a novel mutation, which has not been described yet (HGMD professional 2014.2). “Polyphen2” (14) predicts the consequence of pG785V for the ABCC2 protein as “probably damaging” and “mutation taster” (15) called the variant “disease-causing” At that time, we assumed that the variant represented a pathogenic mutation, but the parents’ missed follow-up with their child, and we failed to outreach to them to get consent for publication. Hence, we could not publish our case report at the time of detection of the novel mutation.

In October 2021, the parents presented to us once again with the patient who was a 21-year young adult female. She got married to her cousin, and she experienced an intermittent deepening of her jaundice during pregnancy with no associated pruritus or dark urine. Her liver biochemistry was within normal values apart from direct hyperbilirubinemia.

She gave birth to a 3.5 kg male baby by normal vertex delivery with uneventful antenatal and perinatal histories. Her baby developed jaundice at the age of 1 week with no history of pallor, blood transfusion, or medications intake.

At the age of 4 weeks, she thought about our medical advice for her newly born jaundiced baby. On assessment, his physical examination was normal apart from mild jaundice with no organomegaly. His workup was assuring with normal abdominal ultrasound, ALT, AST, albumin, prothrombin time/concentration, and GGT with a high total bilirubin of 78 μmol/L and high direct bilirubin of 43 μmol/L, suggesting the diagnosis of DJS.

Sanger sequencing of the p.G785V variant detected in his mother was performed for him, which came out to be positive. The husband was also tested and was heterozygous for the same mutation.

**FIGURE 2:**
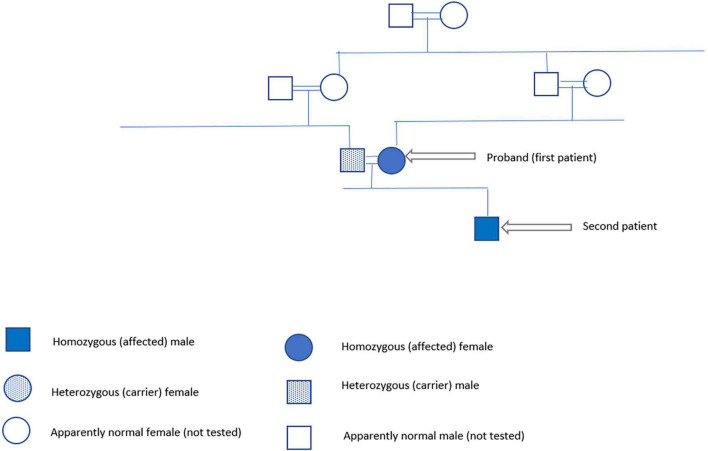
Family pedigree.

In January 2022, we got the consent of the patient and her husband for publishing their family case series. The family pedigree is illustrated in Figure 2.

## Discussion

Dubin-Johnson syndrome is a rare hereditary disease, inherited as autosomal recessive inheritance mainly, but some cases with an autosomal dominant mode of transmission had been reported (10, 11). The first description of the disease was in 1954 (1). It occurs most commonly in Iranian Jews 1:1,300 (16).

Many cases were reported worldwide with the ABCC2 gene mutation (17). Our patient was the first to be detected with c.2273G > T; p.Gly758Val mutation in Exon 18 of the ABCC2 gene, which was in 2014. The patient presented to us 7 years later with her baby having cholestasis, and molecular genetic testing of the same DJS mutation detected in her was tested in her baby and her husband, who were positive to the same mutation in homozygous and heterozygous states, respectively.

Two reports were released from Saudi Arabia, one with a large series of 28 genetically proven cases with DJS (18) and the other one with one non-genetically proven case (19). Twenty-three out of the 28 patients of Al-Hussaini series had the same mutation detected in our family (18).

Patients with DJS have metabolic defect since birth, but it rarely presents in infancy and usually becomes more apparent in the late teens. In our reported two patients and in the series of Al-Hussaini and his colleagues, jaundice started in early infancy.

The first case was missed until the age of 14 years with extensive workup of cholestasis in infancy up to a liver biopsy with more than 20 physicians’ visits afterward, but the diagnosis of DJS was overlooked. This unfortunate course reflects a lack of knowledge about DJS, which should have been the first differential diagnosis of direct hyperbilirubinemia in otherwise normal patients with unremarkable clinical and laboratory workups.

A HIDA scan is a good diagnostic modality for DJS (20, 21), and it was suggestive of the diagnosis in our patient, but molecular genetic testing is the definitive diagnosis. The homozygous variant c.2273G⟩T, pG785V in Exon 18 of the gene ABCC2 gene confirmed the diagnosis in our patient.

## Conclusion

The presence of cholestasis in well-appearing neonates and infants, who have an otherwise normal examination with normal liver biochemical functions and hepatocytes and biliary enzymes, should alert physicians to the possibility of the diagnosis of DJS. A high index of suspicion for DJS in those patients prevents unnecessary lengthy, costly, and invasive workup. Molecular genetic testing of the ABCC2 gene is confirmatory.

## Data Availability Statement

The datasets for this article are not publicly available due to concerns regarding participant/patient anonymity. Requests to access the datasets should be directed to the corresponding author.

## Ethics Statement

The studies involving human participants were reviewed and approved by IRB Committee of Alhada Armed Forces Hospital, Taif, Saudi Arabia. Written informed consent was obtained from the individual(s), and minor(s)’ legal guardian/next of kin, for the publication of any potentially identifiable images or data included in this article.

## Author Contributions

NK diagnosed the patient, set the idea of the study, and designed the study. NK, OS, SA, LS, ME, HA, and AA reviewed literature, drafted the manuscript, and critically analyzed the data. SA, NK, HA, and AA collected patient’s data. All authors reviewed and approved the manuscript for final publication.

## Conflict of Interest

The authors declare that the research was conducted in the absence of any commercial or financial relationships that could be construed as a potential conflict of interest.

## Publisher’s Note

All claims expressed in this article are solely those of the authors and do not necessarily represent those of their affiliated organizations, or those of the publisher, the editors and the reviewers. Any product that may be evaluated in this article, or claim that may be made by its manufacturer, is not guaranteed or endorsed by the publisher.

## Abbreviations

DJS: Dubin-Johnson syndrome.
